# Best Hearing Protectors Ever?

**DOI:** 10.3390/ijerph19042165

**Published:** 2022-02-15

**Authors:** Alberto Behar

**Affiliations:** Department of Psychology, Ryerson University, Toronto, ON M5B 2K3, Canada; alberto.behar@ryerson.ca

The best protectors are those that are worn (Aram Glorig) [1].

The story goes that, around the 8th or 7th century BCE, Ulysses, on his travel back home from the Trojan War, was to sail close to the island of the Sirens. These were dangerous creatures, which lured sailors with their enchanting music and singing voices to shipwreck on the rocky coast of their island. Ulysses wanted to listen to the Sirens without wrecking his ship. He devised a clever scheme: sailors will tie him to the mast, so he could not alter the ship’s course, but the ears of his sailors were plugged up with beeswax, so they could not hear the Siren’s songs (see Figure 1). Those earplugs were so effective that the ship went through while the mates were deaf to the songs and music coming from the island.

Just imagine: 8th century BC, beeswax earplugs that completely attenuated the signal getting into his sailor’s ears! (By the way, the use of beeswax was popular even in the middle of the century).

So, what is my point? I am not advertising the use of beeswax. All I am saying is that hearing protectors have been around for many, many years. What is relatively new is the concept of hearing conservation, a conscious effort to ensure that the hearing of working people does not deteriorate as a result of occupational noise. The concept is prominent in every Health and Safety program. Important resources are invested in assessing hazards in the workplace, in reducing them and, eventually, supplying hearing protection devices to the noise-exposed personnel. In parallel, workers are explained the danger of being exposed to high-level noise for extended periods of time and trained in the proper wear and care of the protecting devices.

At present, protectors have hundreds of types and models: conventional, specialized, active, passive, and different shapes and sizes. Their attenuation is assessed using sophisticated tests, following national and international standards. In other words, there is no lack in devices for use in any kind of noise, under any circumstances. Therefore, it would appear that there is nothing that could be done to improve them!

However, occupational hearing loss is still the most common worker affliction. Many workers’ compensation claims are made worldwide, and many workers end their working life with severe hearing loss.

The reason for this tragedy is simple: people do not wear their protectors or do not do it properly! Not because they do not know how to do it, but because protectors ARE NOT COMFORTABLE. Given, no protective gear is comfortable. The statement applies to all protective gear, from safety shoes to respirators. However, hearing protectors are close to the top of the list of uncomfortable devices, next only to respirators. Consequently, because “ears don’t bleed”, people do not wear them or do not do it properly. Therefore, if education is not sufficient and disciplinary measures do not work, the solution to the problem has to be increased comfort.

Here, we come to an interesting situation: there is no definition for the term “comfort”! Wikipedia defines this as follows: “Comfort (or being comfortable) is a sense of physical or psychological ease, often characterized as a lack of hardship”. This is a curious way of defining something, through a negative (“…lack of…”) characteristic. Therefore, we are talking about something that has no definition!

We do not have methods for measuring comfort. There are no standards for measurement. The reason for this apparent desert in knowledge of comfort is the inherent individuality that makes it so personal. It is definitely a very difficult subject to deal with. However, it has to be tackled once and for all to begin working on a solution.

Until we define what a comfortable protector is, and manufacture one, the “best protector ever” will, unfortunately, continue to be a pie in the sky!

## Figures and Tables

**Figure 1:**
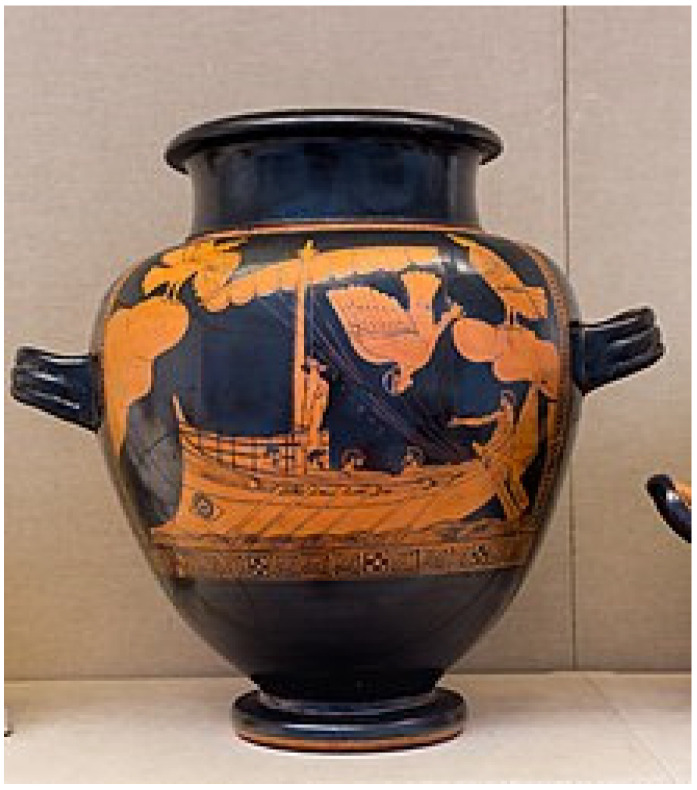
Odysseus and the Sirens, eponymous vase of the Siren Painter, c. 475 BC (From Wikipedia https://en.wikipedia.org/wiki/Siren_(mythology)#/media/File:Siren_Painter_ARV_289_1_Odysseus_and_the_Sirens_-_three_erotes_(02).jpg accessed on 27 January 2022).
